# Supplementary figures and images for: A metric for evaluating biological information in gene sets and its application to identify co-expressed gene clusters in PBMC
Source: PLoS Comput Biol. 2021 Oct 6;17(10):e1009459. doi: 10.1371/journal.pcbi.1009459 (PMC8523066; doi:10.1371/journal.pcbi.1009459)

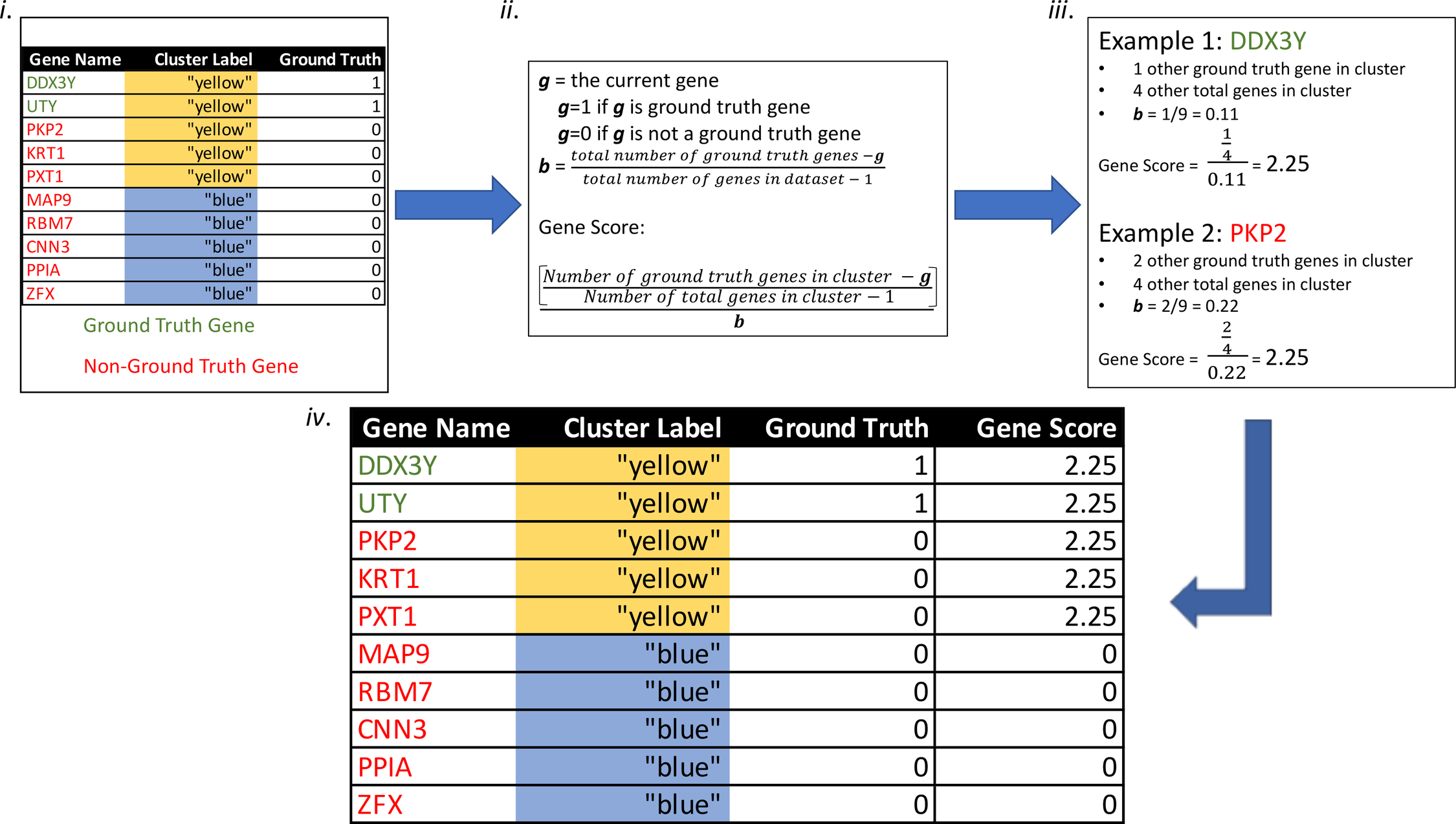

Supplement: S1 Fig — i.) The GECO metric requires a list of genes, a label indicating to which cluster each gene has been assigned, and a Boolean value indicating if the gene is a ground truth gene. ii.) Each gene is then assigned a gene score based on the likelihood of that gene being a ground truth gene based on the makeup of the cluster to which the gene belongs. iii.) Two sample genes scored, one a ground truth gene and one a regular gene, to illustrate the scoring process. iv.) The gene scores are stored to later be used in the final calculation of the GECO metric. (TIF) [file pcbi.1009459.s001.tif]

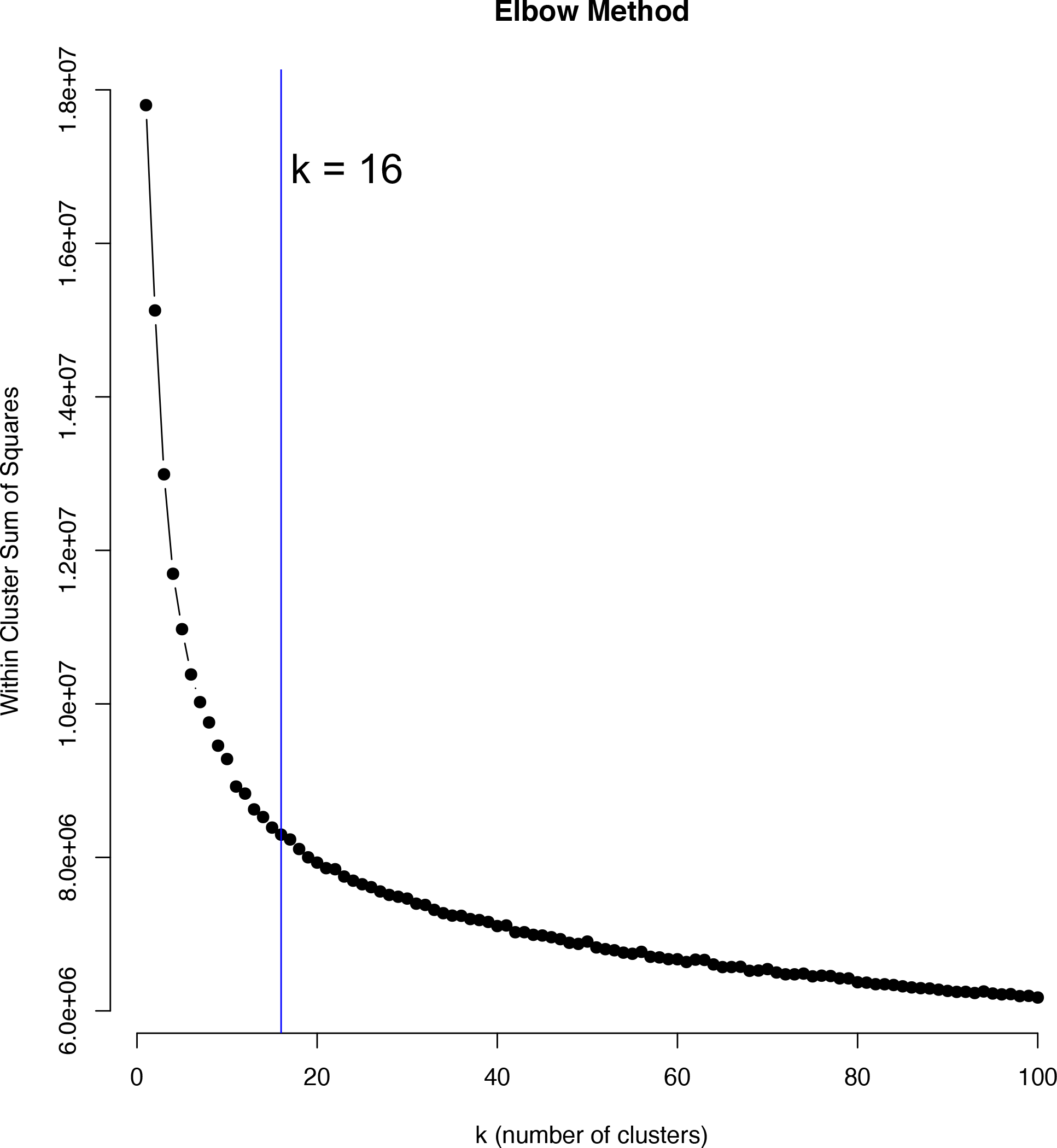

Supplement: S2 Fig — The results of the “elbow method” to determine the optimal number of clusters. A k-value of 16 was chosen based on inspection of the graph, however, no defined inflection point could be definitively identified. (TIF) [file pcbi.1009459.s002.tif]

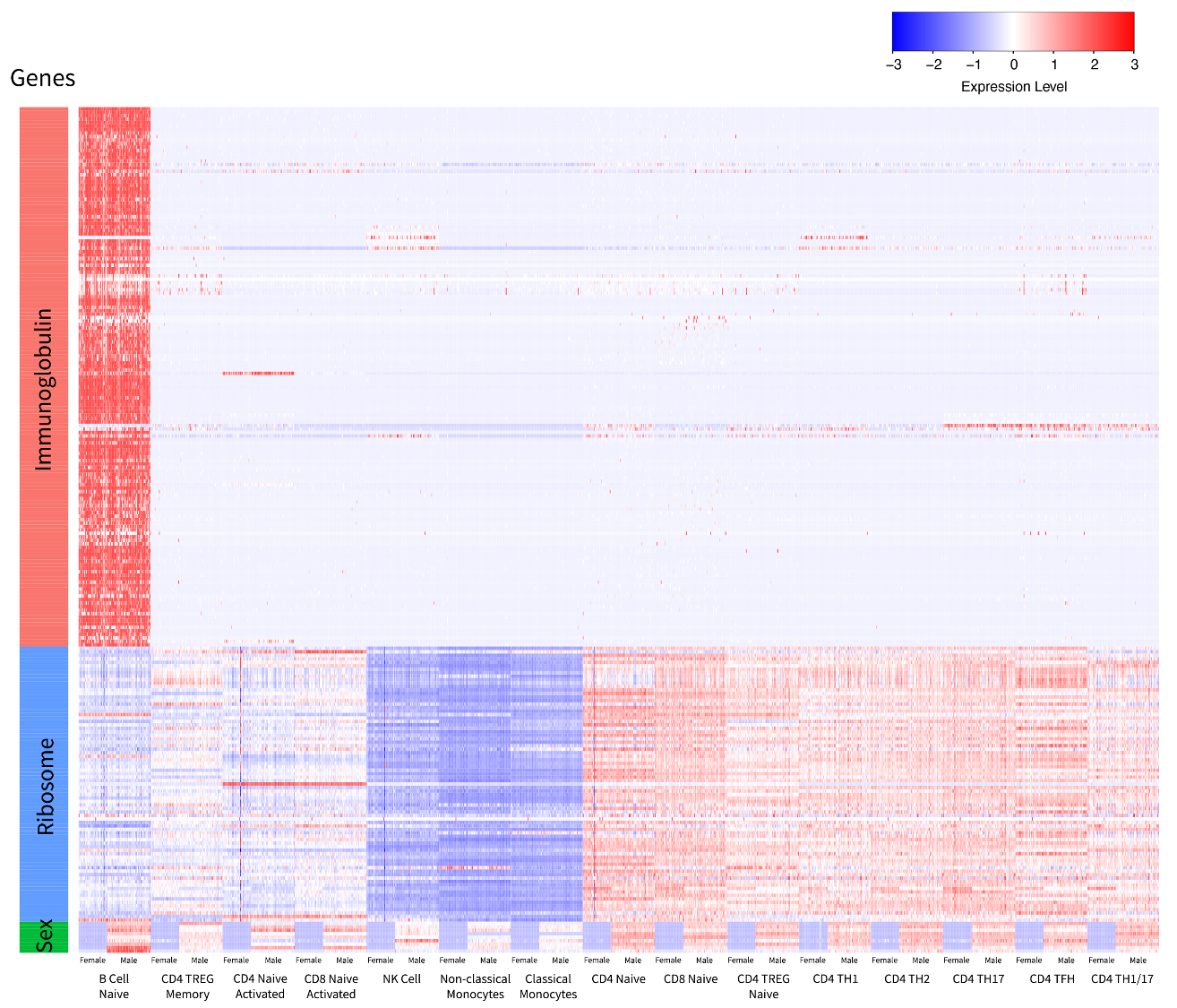

Supplement: S3 Fig — The co-expression of our three ground truth gene sets as visualized using a heatmap. The values used are RNA-seq expression levels after TPM and Z-score normalization. A range of values, from -3 to 3, were used to visualize the heatmap with any values outside of that range reassigned to the nearest threshold. The ground truth sets are indicated along the y-axis. Red indicates positive scores while blue indicates negative scores. (TIF) [file pcbi.1009459.s003.tif]

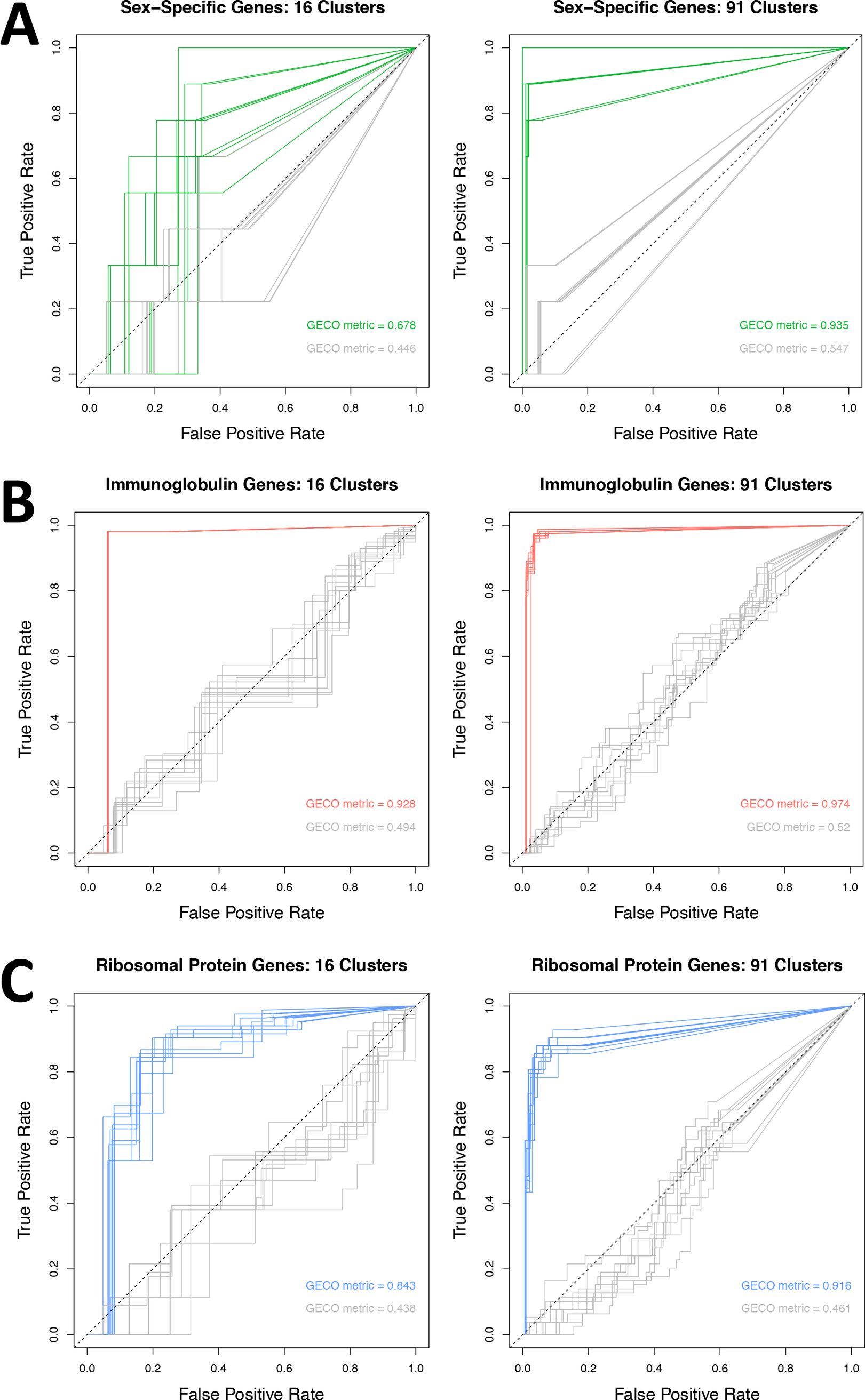

Supplement: S4 Fig — ROC plots showing the increase in cluster quality for the given ground truth gene set when increasing from k = 16 to k = 91. The GECO metric for each set is shown in their respective color and represent the mean cluster quality score. A. Sex-specific ground truth gene set: Each green line represents the GECO score for a single iteration at the given k-value and the grey lines are iterations with random genes selected and scored as pseudo sex-specific genes. B. Immunoglobulin ground truth gene set: Each red line represents the GECO score for a single iteration at the given k-value and the grey lines are iterations with random genes selected and scored as pseudo immunoglobulin genes. C. Ribosomal Protein ground truth gene set: Each blue line represents the GECO score for a single iteration at the given k-value and the grey lines are iterations with random genes selected and scored as pseudo ribosomal protein genes. (TIF) [file pcbi.1009459.s004.tif]

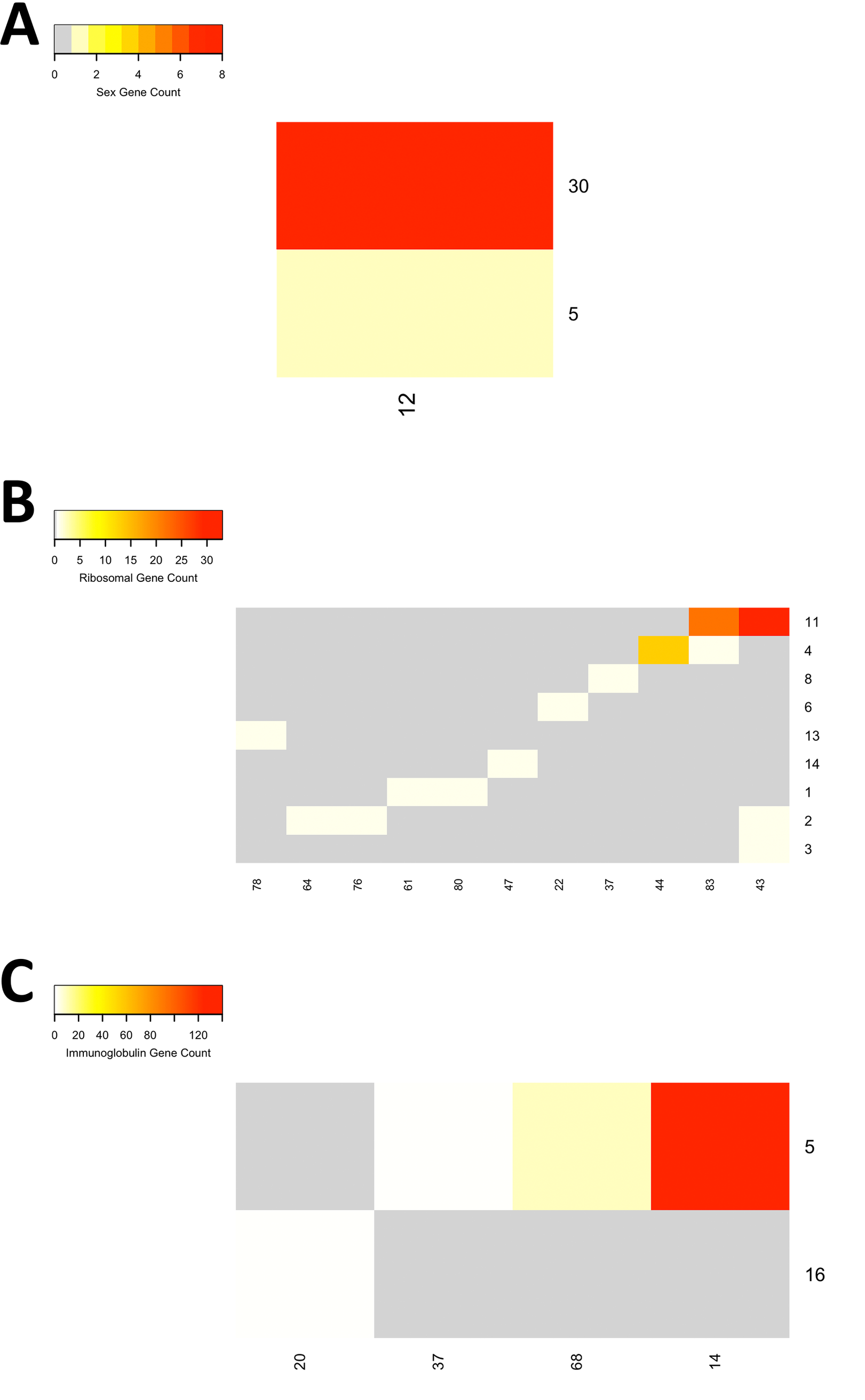

Supplement: S5 Fig — The x-axis shows the cluster numbers from the k91 DICE clusters, while the y-axis shows the cluster numbers from the WGCNA clusters. A. The overlap of genes from the sex-specific (Y-chromosome) ground truth set between the k91 DICE clusters and the WGCNA clusters. All sex-specific genes were found within a single k91 DICE cluster. B. The overlap of genes from the ribosomal ground truth set between the k91 DICE clusters and the WGCNA clusters. C. The overlap of genes from the immunoglobulin ground truth set between the k91 DICE clusters and the WGCNA clusters. (TIF) [file pcbi.1009459.s005.tif]

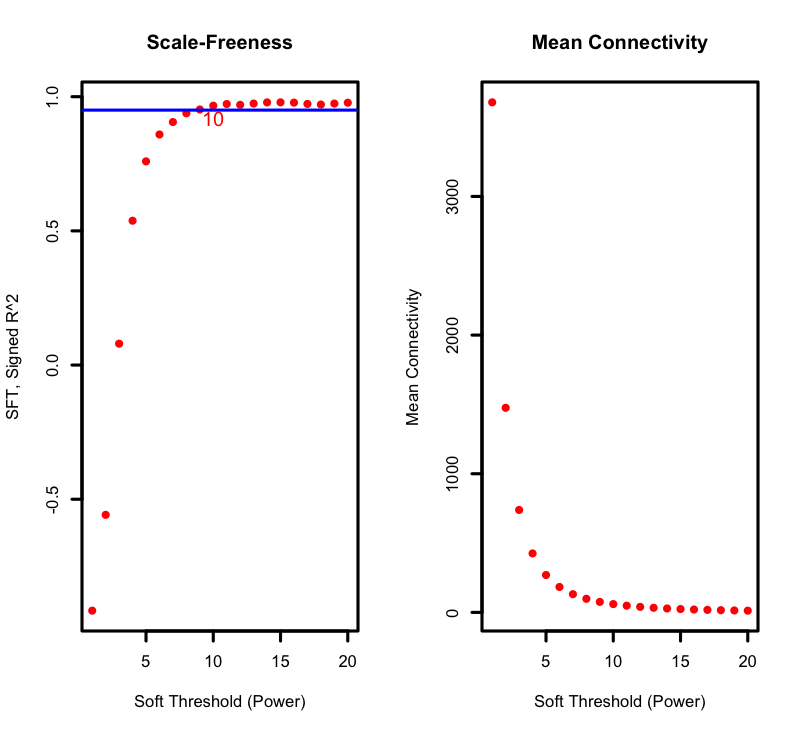

Supplement: S6 Fig — The WGCNA R package requires a user-defined soft-threshold (referred to as the “power” in the function call) based on visually observing the above graph; specifically, the scale-freeness. The first value above the threshold suggested by the WGCNA authors (0.95) was chosen as indicated by the graph. Combined with the negative slope indicated in the mean connectivity graph, we can assume that we have a scale-free network as required by WGCNA. (TIF) [file pcbi.1009459.s006.tif]
