# Supplementary material for: A metric for evaluating biological information in gene sets and its application to identify co-expressed gene clusters in PBMC
Source: PLoS Comput Biol. 2021 Oct 6;17(10):e1009459. doi: 10.1371/journal.pcbi.1009459 (PMC8523066; doi:10.1371/journal.pcbi.1009459)
Supplement: S1 Table — The ground truth genes contained within each ground truth set. (RTF) [file pcbi.1009459.s007.rtf]

IMMUNOGLOBULIN GENESEnsembl_ID	Gene_NameENSG00000211592.8	IGKCENSG00000211593.2	IGKJ5ENSG00000211594.2	IGKJ4ENSG00000211595.2	IGKJ3ENSG00000211597.2	IGKJ1ENSG00000211598.2	IGKV4-1ENSG00000211599.2	IGKV5-2ENSG00000211611.2	IGKV6-21ENSG00000211623.2	IGKV2D-26ENSG00000211625.2	IGKV3D-20ENSG00000211632.4	IGKV3D-11ENSG00000211637.2	IGLV4-69ENSG00000211638.2	IGLV8-61ENSG00000211639.2	IGLV4-60ENSG00000211640.4	IGLV6-57ENSG00000211642.3	IGLV10-54ENSG00000211643.2	IGLV5-52ENSG00000211644.3	IGLV1-51ENSG00000211645.2	IGLV1-50ENSG00000211647.1	IGLV5-48ENSG00000211648.2	IGLV1-47ENSG00000211649.3	IGLV7-46ENSG00000211650.2	IGLV5-45ENSG00000211651.3	IGLV1-44ENSG00000211652.2	IGLV7-43ENSG00000211653.2	IGLV1-40ENSG00000211654.2	IGLV5-37ENSG00000211655.3	IGLV1-36ENSG00000211658.2	IGLV3-27ENSG00000211659.2	IGLV3-25ENSG00000211660.3	IGLV2-23ENSG00000211661.2	IGLV3-22ENSG00000211662.2	IGLV3-21ENSG00000211663.2	IGLV3-19ENSG00000211664.3	IGLV2-18ENSG00000211665.3	IGLV3-16ENSG00000211666.2	IGLV2-14ENSG00000211667.3	IGLV3-12ENSG00000211668.2	IGLV2-11ENSG00000211669.3	IGLV3-10ENSG00000211670.2	IGLV3-9ENSG00000211672.2	IGLV4-3ENSG00000211673.2	IGLV3-1ENSG00000211676.2	IGLJ2ENSG00000211677.2	IGLC2ENSG00000211678.2	IGLJ3ENSG00000211679.2	IGLC3ENSG00000211685.3	IGLC7ENSG00000211890.4	IGHA2ENSG00000211891.6	IGHEENSG00000211892.4	IGHG4ENSG00000211893.4	IGHG2ENSG00000211895.5	IGHA1ENSG00000211896.7	IGHG1ENSG00000211897.9	IGHG3ENSG00000211898.7	IGHDENSG00000211899.9	IGHMENSG00000211900.2	IGHJ6ENSG00000211904.2	IGHJ2ENSG00000211905.1	IGHJ1ENSG00000211907.1	IGHD1-26ENSG00000211909.1	IGHD5-24ENSG00000211911.1	IGHD3-22ENSG00000211912.1	IGHD2-21ENSG00000211914.1	IGHD6-19ENSG00000211917.1	IGHD3-16ENSG00000211918.1	IGHD2-15ENSG00000211920.1	IGHD6-13ENSG00000211921.1	IGHD5-12ENSG00000211923.1	IGHD3-10ENSG00000211924.1	IGHD3-9ENSG00000211925.1	IGHD2-8ENSG00000211930.1	IGHD3-3ENSG00000211931.1	IGHD2-2ENSG00000211933.2	IGHV6-1ENSG00000211934.3	IGHV1-2ENSG00000211935.3	IGHV1-3ENSG00000211937.3	IGHV2-5ENSG00000211938.2	IGHV3-7ENSG00000211941.3	IGHV3-11ENSG00000211942.3	IGHV3-13ENSG00000211943.2	IGHV3-15ENSG00000211945.2	IGHV1-18ENSG00000211946.3	IGHV3-20ENSG00000211947.2	IGHV3-21ENSG00000211949.3	IGHV3-23ENSG00000211950.2	IGHV1-24ENSG00000211951.2	IGHV2-26ENSG00000211952.3	IGHV4-28ENSG00000211955.2	IGHV3-33ENSG00000211956.2	IGHV4-34ENSG00000211957.2	IGHV3-35ENSG00000211958.2	IGHV3-38ENSG00000211959.2	IGHV4-39ENSG00000211961.3	IGHV1-45ENSG00000211962.2	IGHV1-46ENSG00000211964.3	IGHV3-48ENSG00000211965.4	IGHV3-49ENSG00000211966.2	IGHV5-51ENSG00000211967.3	IGHV3-53ENSG00000211968.3	IGHV1-58ENSG00000211970.3	IGHV4-61ENSG00000211972.2	IGHV3-66ENSG00000211973.2	IGHV1-69ENSG00000211976.2	IGHV3-73ENSG00000223350.2	IGLV9-49ENSG00000223648.4	IGHV3-64ENSG00000224041.3	IGKV3D-15ENSG00000224373.3	IGHV4-59ENSG00000224650.2	IGHV3-74ENSG00000225523.2	IGKV6D-21ENSG00000225698.3	IGHV3-72ENSG00000225825.1	IGHD6-25ENSG00000227108.1	IGHD1-14ENSG00000227196.1	IGHD4-23ENSG00000227800.1	IGHD4-17ENSG00000228131.1	IGHD6-6ENSG00000231475.3	IGHV4-31ENSG00000232216.1	IGHV3-43ENSG00000232543.2	IGHD4-11ENSG00000233655.1	IGHD4-4ENSG00000236170.1	IGHD1-1ENSG00000236597.1	IGHD7-27ENSG00000237020.1	IGHD1-20ENSG00000237197.1	IGHD1-7ENSG00000239571.1	IGKV2D-30ENSG00000239819.2	IGKV1D-8ENSG00000239855.1	IGKV1-6ENSG00000239951.1	IGKV3-20ENSG00000240041.1	IGHJ4ENSG00000240382.3	IGKV1-17ENSG00000240671.4	IGKV1-8ENSG00000240864.3	IGKV1-16ENSG00000241244.1	IGKV1D-16ENSG00000241294.1	IGKV2-24ENSG00000241351.3	IGKV3-11ENSG00000241566.1	IGKV2D-24ENSG00000241755.1	IGKV1-9ENSG00000242472.1	IGHJ5ENSG00000242580.1	IGKV1D-43ENSG00000242766.1	IGKV1D-17ENSG00000242887.1	IGHJ3ENSG00000243063.1	IGKV3-7ENSG00000243238.1	IGKV2-30ENSG00000243264.2	IGKV2D-29ENSG00000243290.3	IGKV1-12ENSG00000243466.1	IGKV1-5ENSG00000244437.1	IGKV3-15ENSG00000244575.3	IGKV1-27ENSG00000251546.1	IGKV1D-39ENSG00000270550.1	IGHV3-30ENSG00000274576.2	IGHV2-70ENSG00000276566.1	IGKV1D-13ENSG00000276775.1	IGHV4-4ENSG00000278196.3	IGLV2-8RIBOSOMAL PROTEIN GENESEnsembl_ID	Gene_NameENSG00000008988.9	RPS20ENSG00000037241.7	RPL26L1ENSG00000071082.10	RPL31ENSG00000083845.8	RPS5ENSG00000089009.15	RPL6ENSG00000089157.15	RPLP0ENSG00000100316.15	RPL3ENSG00000105193.8	RPS16ENSG00000105372.6	RPS19ENSG00000105640.12	RPL18AENSG00000108107.14	RPL28ENSG00000108298.9	RPL19ENSG00000109475.16	RPL34ENSG00000110700.6	RPS13ENSG00000112306.7	RPS12ENSG00000114391.12	RPL24ENSG00000115268.9	RPS15ENSG00000116251.9	RPL22ENSG00000118181.10	RPS25ENSG00000122026.10	RPL21ENSG00000122406.12	RPL5ENSG00000124614.13	RPS10ENSG00000125691.12	RPL23ENSG00000130255.12	RPL36ENSG00000131469.12	RPL27ENSG00000134419.15	RPS15AENSG00000136942.14	RPL35ENSG00000137154.12	RPS6ENSG00000137818.11	RPLP1ENSG00000138326.18	RPS24ENSG00000140988.15	RPS2ENSG00000142534.6	RPS11ENSG00000142541.16	RPL13AENSG00000142676.12	RPL11ENSG00000142937.11	RPS8ENSG00000143947.13	RPS27AENSG00000144713.12	RPL32ENSG00000145425.9	RPS3AENSG00000145592.13	RPL37ENSG00000146223.14	RPL7L1ENSG00000147403.16	RPL10ENSG00000147604.13	RPL7ENSG00000148303.16	RPL7AENSG00000149273.14	RPS3ENSG00000156482.10	RPL30ENSG00000161016.17	RPL8ENSG00000161970.12	RPL26ENSG00000162244.10	RPL29ENSG00000163682.15	RPL9ENSG00000163923.9	RPL39LENSG00000164587.11	RPS14ENSG00000165502.6	RPL36ALENSG00000166441.12	RPL27AENSG00000170889.13	RPS9ENSG00000171858.17	RPS21ENSG00000171863.12	RPS7ENSG00000172809.12	RPL38ENSG00000174444.14	RPL4ENSG00000174748.18	RPL15ENSG00000177600.8	RPLP2ENSG00000177954.11	RPS27ENSG00000182774.10	RPS17ENSG00000182899.14	RPL35AENSG00000185088.13	RPS27LENSG00000186468.12	RPS23ENSG00000188846.13	RPL14ENSG00000197728.9	RPS26ENSG00000197756.9	RPL37AENSG00000197958.12	RPL12ENSG00000198034.10	RPS4XENSG00000198242.13	RPL23AENSG00000198755.10	RPL10AENSG00000198918.7	RPL39ENSG00000213741.9	RPS29ENSG00000229117.8	RPL41ENSG00000231500.6	RPS18ENSG00000233927.4	RPS28ENSG00000241343.9	RPL36AENSG00000265681.7	RPL17SEX-SPECIFIC GENESEnsembl_ID	Gene_NameENSG00000012817.15	KDM5DENSG00000067048.16	DDX3YENSG00000067646.11	ZFYENSG00000114374.12	USP9YENSG00000129824.15	RPS4Y1ENSG00000154620.5	TMSB4YENSG00000183878.15	UTYENSG00000198692.9	EIF1AYENSG00000280969.1	RPS4Y2
