# Supplementary material for: A metric for evaluating biological information in gene sets and its application to identify co-expressed gene clusters in PBMC
Source: PLoS Comput Biol. 2021 Oct 6;17(10):e1009459. doi: 10.1371/journal.pcbi.1009459 (PMC8523066; doi:10.1371/journal.pcbi.1009459)
Supplement: Attachment — Submitted filename: PLOS_reviewer_reply.docx [file pcbi.1009459.s015.docx]

**Reviewer #1**: **Bennett et al described a really clever metric for clustering which they call GECO (Ground truth and evaluation of clustering outcomes). They used transcriptomic signatures such as ribosomal signatures, male gender-specific signature and an immunoglobulin signature to measure how well certain clustering methods cluster these genes together. I think the method itself is really clever. I have a few comments regarding the manuscript.**

**Mayor issues:
Probably the authors have forgotten to mention that they used the logarithmic count of the expression values. Using the expression values directly in a k-mean clustering will make the absolute values dive the clustering and not which genes are co-expressed, have similar gene expression pattern in the samples. I have read that they used the TPM normalized Z scores, but I am not sure whether the Z scores are normalized gene-wise or sample wise. They should be normalized gene-wise to be comparable. Please add this or use log(TPM) counts, because those are closer to a normal distribution, where k means is applied.**

The reviewer is correct, using the raw read counts without normalization would not produce meaningful gene clusters. We thank the reviewer for catching the omission and can confirm that the Z-scores were normalized in a gene-wise fashion. This has been corrected and included on line 382.

**My other issue is the comparison with WGCNA. The authors optimized only one parameter of the WGCNA analysis (deepSplit parameter). WGCNA is sensitive to the power of the correlations are used. Please show the graphs of the scale-freeness of choosing power 10 as a supplementary figure.**

We thank the reviewer for bringing this to our attention and have added a comment in the methods describing the selection of our “power” variable at lines 520-522. We have also added a new supplementary figure showing both the scale-freeness and the mean connectivity. This is now referred to as Supplementary Figure 6.

**Also in WGCNA what was the actual input data - Z score normalized gene expression or TPM?**

The reviewer has correctly identified the lack of detail regarding the data supplied to WGCNA. The input data to WGCNA was the TPM and Z-score normalized data. This has been added at line 516-517.

**I suggest adding a heatmap that compares the WGCNA, k-means and k-91 DICE clustering similarity using the various datasets - overlap percentage in the squares of the heatmap.**

This is a good suggestion, and we have prepared heatmaps – one for each ground truth gene set – comparing the WGCNA and the k91 DICE clusters (done by k-means) as Supplementary Figure 5. It clearly shows that the ground truth gene clustering overlaps between the methods.

**On the gene expression analysis what background was used? Please describe it in the methods.**

The reviewer is correct, we did not mention our background for the GO and KEGG enrichments. We apologize and have included this information within the methods section at lines 499-500 and 507-508.

**Minor:
Please check whether all acronyms are defined. I am not sure that TB is defined in the introduction or in the results.**

The reviewer is correct. We have checked the manuscript again to make sure all acronyms have been defined before use. TB is the acronym for Tuberculosis which was accidentally omitted. We have made the appropriate statements to define TB in the manuscript at line 195 and thank the reviewer for their comment.

**Reviewer #2: The main problem in analyzing transcriptomics data is that the number of independent samples is typically much lower (<100)
than the number of genes whose expression is quantified, particularly for the single cell RNA-seq.
The authors to design a novel metric for the evaluation of clustering of genes based on the gene expression profiles.
In all, the manuscript is well-organized and easy to follow. However, I found some many problems:

1. The authors claim that the proposed GECO metric is computational efficient, robust and superior to the current alternatives.
As shown the Section GECO metric (Line: 137), the proposed method only exploit the overlapping genes between the predicted cluster
and ground truth one, which is very similar to the classic metric, such as Jaccard index, and hypergeometric test. Thus, I doubt
the novelty of metric. I notice that the subtle difference exists, how the gentle modification of the available alternatives may not
be very interesting.**

The reviewer is correct in connecting the GECO metric to existing metrics like the Jaccard index and/or a hypergeometric test. These metrics were certainly inspirations for creating the GECO metric and represent examples of statistical techniques that employ a similar approach as GECO. However, both the Jaccard index and a hypergeometric test lack the ability to directly compare two sets of clusters with different numbers of clusters. That is, if we have two sets of clusters – one with ‘n’ total clusters and one with ‘m’ total clusters (where n ≠ m) – we cannot use the Jaccard index or a hypergeometric test to directly compare them. We can use these methods to assess the clusters individually, but the resultant measurements cannot be compared to each other. This is because both methods require a count of “successes”; the number of genes that are correctly grouped into a single “correct” cluster. When two different sets of clusters are compared with differing numbers of clusters in each, counting the “correct” number of genes becomes impossible. There is no one-to-one mapping that can be done between the clusters of one set to another, and therefore, the results of either the Jaccard test or a hypergeometric test will hold only for a single cluster set. The GECO metric allows us to perform this kind of comparison because it looks for the grouping of ground truth genes across all clusters, without the need to designate a single “correct” cluster. This is an incredibly important aspect of the GECO metric that we did not accurately convey, and we thank the reviewer for bringing this to our attention. We have included a new section in the introduction to mention this concern which can be found at lines 89-94.

**2. One issue really confused me is that the authors adopt k-means to validate the performance of the proposed metric. Actually, k-means is
criticized for the instability, sensitivity to the initial solution, and low accuracy. There are some many algorithms can be used, such
as Hierarchical clustering, spectral clustering, principal component analysis, and nonnegative matrix factorization, which significantly
outperforms k-means. Why do not you take these ones?**

The reviewer is correct, there exist many clustering approaches that offer better end results when used by themselves to generate gene clusters. However, our focus was on the development of a metric to evaluate clusters and not on the method of clustering itself. We needed a method that allowed us to generate many clustering outcomes with slightly varying numbers of clusters. K-means gave us the clustering data that we needed to develop the GECO metric. In addition, k-means represents one of the most widely used and well-studied methods of clustering and was selected as an adequate choice to use as a baseline. When creating the GECO R package, we continued to use k-means because it offered the most control over the number of resultant clusters (which is a property we wanted to maintain) and because we had shown that k-means produces meaningful clusters when used with the metric. Any set of gene clusters generated by any method of clustering could be evaluated using the metric, as described when we used GECO to evaluate our WGCNA clusters. We have now pointed out that k-means was chosen for convenience in the discussion, and that many other methods such as spectral clustering, principal component analysis, and nonnegative matrix factorization are available and expected to be superior.

**3. In the experiments, the authors only employ three gene sets to validate the performance, which is insufficient to validate the performance.**

The reviewer is correct in expressing that three gene sets would be insufficient for performing a strict statistical analysis between metrics. Our choice to include these three was not based on proving a statistical difference between metrics, but to show how the performance of the GECO metric varies in different biological contexts. The three gene sets were chosen to represent biologically distinct drivers of gene expression patterns. The ribosomal protein gene set was a set of genes that should be co-expressed based on cellular structure (large and small ribosomal subunits must be co-expressed.) The immunoglobulin gene set will be co-expressed in a cell-type specific manner, identifying B-cell clusters. Finally, the Y-chromosome sex-specific gene set identifies a strong biological signal that will be picked up by even the most indelicate clustering approach. These three gene sets thus represent very different biological processes, but all three similarly capture a fundamental trend in the data.

To expand beyond the three gene sets, we have now analyzed all gene sets included in the molecular signatures database (MSigDB) “Hallmark genes” section. This database contained an additional 50 gene sets which are commonly used for gene set enrichment. This resulted in an additional supplementary table detailing the performance of the k91 DICE clusters in identifying the gene sets contained in MSigDB as measured by the GECO metric. This was a valuable addition to the manuscript, and we thank the reviewer for their suggestion. The supplementary table is called Supplementary Table 6 and the analysis of the MSigDB Hallmark gene sets have been detailed in a new section in the Results at lines 254-278.

**Furthermore, the biological story is not very interesting.
Based on the consideration above, I cannot recommend it to be published.**

We are grateful for the consideration and the time that the reviewer has volunteered. Their suggestions and concerns have been noted and have been used to make valuable edits and additions. However, we respectfully disagree with the reviewer’s conclusion that the results are not interesting, as the methodological insights presented justify the use of much more fine-grained gene clusters for analysis than what is current practice. So, while we agree that there is no major biological story in this paper, we believe that our findings will help in obtaining such discoveries.

**Reviewer #3: The authors of this manuscript highlight and address important drawbacks of current gene cluster/module identification from gene expression data - namely that unbiased reduction of gene expression into clusters/modules may identify ones that discriminate among samples based on confounding factors, and that there are no methods to determine the biologically optimal number of clusters/modules. The authors note the existing disconnect between the technical principles at play in determining “optimal” clustering parameters from a mathematical perspective, and the necessity to cluster genes into clusters that illustrate true biological signal in gene expression. They propose and describe a method that uses sets of consistently/canonically co-expressed sets of genes to inform and “ground-truth” the module building process; potentially improving module building from new datasets by aiding the user in selecting technical parameters such that resulting modules are optimized for their biological relevance. From this method, they develop a set of 91 genesets that can be used in particular for immune cell type specific expression profiling experiments. Overall, their approach is sound and the method represent a valuable contribution to the field. However, I have several suggestions to clarify and potentially strengthen the manuscript.

Major Comments:
The manuscript would be strengthened by acknowledging/exploring potential drawbacks of the proposed method (there are currently few caveats mentioned). I.e., could this method risk simply confirming already described expression profiles/clusters by using those existing gene sets to “ground truth” findings?**

The reviewer is correct in that more information about strategies to avoid overfitting are required. There is a risk that the metric will indicate a single clustering configuration as optimal based on one ground truth set. Ultimately the user has to decide how many ground truth sets to use and which ones are most informative for their dataset. It is conceivable that a user may input a single co-expressed gene set and either omit further sets or focus on only one of the gene sets to come to a conclusion about the optimal clustering. The reviewer has touched on an important point and this concern has resulted in an additional section being added to the Discussion found at lines 325-337.

**Similarly, could it risk fine tuning mathematical parameters until finding a predefined signal for a given dataset (which presumably could occur if disease specific gene sets were used as the “ground truth”)? How does the method avoid these types of issues? While I do not believe the method necessarily would result in these drawbacks, the manuscript should head off these concerns by discussing them.**

The reviewers concern is definitely warranted. It can be easy for unsupervised learning methods (such as those involved in clustering) to fall prey to inadvertently optimizing a solution to a particular problem instead of to a more general concern. In our case, the inclusion of multiple ground truth sets representing highly biologically distinct concepts should protect against overfitting. With multiple known co-expressed gene sets, we can safely expect the majority of genes in a gene set to cluster together if the clustering has produced biologically meaningful clusters. In the case where one gene set indicates a vastly different optimal clustering configuration than all others, as the reviewer mentioned, this has not occurred. At that point, the remaining sets should serve to bring attention to this imbalance so that the user can make a decision about which sets to prioritize and how best to perform their clustering based on their needs and the dataset. Overall, this concern does deserve more consideration in the manuscript and has been mentioned in the new paragraph added to the Discussion section (and specifically at lines 329-334.)

**To this end, further defining guidelines for the selection/curation of appropriate ground-truth gene sets would be valuable (and standardized way to first test for appropriate co-expression of ground-truth gene sets within the data at hand). To what extent does responsibility fall purely on the user to define/test these ground-truth gene sets?**

The reviewer is absolutely correct; the selection and curation of ground truth genes is one of the fundamental steps that will determine the success or failure of the metric. More information regarding the ground truth genes, how they were selected, and methods for users to select their own should be included and will be added into the manuscript. These concerns have been mentioned in the new paragraph found in the Discussion section on lines 334-337.

**While the manuscript is generally well written and clearly worded, there are certain areas that lack clarity. For example it is often difficult to discern which underlying test dataset the authors are discussing, as it varies from section to section within the results and is sometimes not stated. Furthermore, the paper presents a substantial amount of methods information in the results section, which makes is difficult to readily gain comprehensive understanding of their process while reading through the manuscript. Some of the results section would be more appropriately placed in the introduction or methods, and some restructuring suggestions are made below.**

We thank the reviewer for their thoughts on how to improve the format of the manuscript and have adjusted multiple aspects of the manuscript as detailed in the following comments.

**The current manuscript focuses on 3 ground truth gene sets that represent canonical biology. The authors extend the work to show the potential value with a more context specific dataset using TB gene set signatures as ground truth comparisons. To the earlier point about appropriate selection of ground truth gene sets, the authors may want to consider extending this to other signatures that have been well characterized – eg. interferon stimulated genes, canonical signaling pathways, MSigDB Hallmark gene sets. This may strengthen the manuscript for a broad audience.**

This was an excellent suggestion by the reviewer. We first tried to look at the interferon-stimulated genes and how they were captured by the k91 DICE clusters. They were fairly evenly dispersed across the clusters, leading to a low GECO metric score. We then looked at the MSigDB Hallmark gene sets and decided that they were both a better curated gene set than the interferon-stimulated genes (found through a literature search) and that the variability of the gene sets was more useful for characterizing our k91 DICE clusters. As a result, an additional supplementary table has been created detailing the performance of the k91 DICE clusters in identifying the gene sets contained in MSigDB. The supplementary table is called Supplementary Table 6 and the analysis of the MSigDB Hallmark gene sets have been detailed in a new section in the Results at lines 254-278.

Also of note, this suggestion led to a realization that the R package lacked a simple function to take a set of clusters and a set of ground truth genes and score them. Prior to this, the R package provided methods to take a dataset, cluster it, and score it. The straight-forward application of the metric to a precomputed set of clusters needed to be included. We have added this to the R package and once again thank the reviewer for their suggestion which brought about a significant improvement to the GECO package.

**Evaluation and comparison of two clustering methods (k-means and WGCNA) is an important asset of this manuscript. However, WGCNA is not mentioned until the middle of the methods section (and there seems like a bit of an afterthought). The authors later include valuable discussion comparing the two methods and assessing the potential utility of the GECO approach into both. Including some of this discussion in the introduction as it increases the relevance of the manuscript and provides additional justification for the new method proposed.**

This is good suggestion from the reviewer. WGCNA is at the forefront of clustering efforts and our comparisons to WGCNA clusters were a valuable part of our methods and should have been mentioned sooner in the manuscript. Earlier mentions of WGCNA have been added at lines 82-85 of the introduction.

**As noted above, the manuscript as presented will benefit from some restructuring. Currently the methods and results section are somewhat poorly distinguished, with many relevant methods details not given until the results section (e.g. assessment of ground-truth gene sets for co-expression in the analysis dataset, multiple iteration of k-means clustering to account for stochasticity, gene set enrichment analysis). This leaves the reader desiring more detail that is then not given until later in the manuscript. The overview given under “Establishment of the GECO Metric” in the results section would be a helpful outline of the methods if included at the end of the introduction or beginning of the methods section. I would recommend beginning the methods section with this sort of brief but comprehensive overview of the analytic process (expanding conceptual figure 1 and citing it in this section), before giving specific methodological detail in each step. This journal generally requests a “results & discussion before methods” format, and the authors could consider restructuring their manuscript in this way.**

The reviewer is correct about the expectations of PLOS and we are grateful for the formatting suggestions. We have reordered the manuscript to conform with PLOS standards and guidelines.

**Methods - GECO Metric This section needs work adding clarity to the description of how the metric is derived. It may be clearer to define the mathematical calculations first, and then describe their intuitive interpretation. This section could also be better organized to avoid repeating the calculations. The calculations presented in Figure 1 are a much more intuitive way of describing the approach than is given in the text. I would make the description of the calculation more like that given in the figure. Figure 1 could be cited sooner, in the methods section as it is helpful to understand the workflow. The ROC method used to generate the GECO score should be better explained, both in terms of what this method technically calculates, and an intuitive explanation of what this information tells the user (ie, why is this the optimal way to derive the metric). I believe that this is more or less and integrative method to evaluate the ability to predict co-occurrence of genes from a ground-truth set within modules, but this lacks clarity. It seems that there should also be a description of how gene scores are used to generate predictions on which the ROC curves are fitted. Figure 1 helps explain how the gene scores described in this section are used to produce the GECO metric via ROC (though it seems a prediction step should be added), reference it.**

We agree with the reviewer that this section needed to be rearranged. We have moved the mathematical portion of the section prior to the conceptual explanation as suggested by the reviewer. We have made additional efforts to avoid repetition. Regarding the two methods of explaining the calculations, we agree that the figure presents a more easily digestible introduction, and we have tried to make the changes to this section reflect the approach that was used in the figure. Additional explanations regarding ROC and tying that into scoring along with significant rearrangement of the section overall can be found at lines 405-484.

**Minor comments/Line Notes:

L277-288 Description of evaluating the GECO metric is described first and in detail only for the ribosomal gene set. This is somewhat confusing to the reader and would be clearer to describe the general process & then state that it was conducted for each of three gene sets and present the results.**

We thank the reviewer for their comment and have taken another look at our presentation of the metric. We agree that the general approach would better translate to the process used in the metric, however, we feel that without the individual example, the metric process becomes a bit convoluted. Using the ribosomal protein gene set by itself presents the reader with an overview for each step in the metric process that a general process could not achieve.

**L301 – the listed k values for WGCNA “49 and 46” are in reverse order of what they should be for deepsplit 3 vs 4.**

The reviewer is correct, this has been fixed in the manuscript at line 181.

**L328 Clarify that the “74-gene TB signature” is derived from the Burel dataset. Is this not problematic to ground-truth modules built from the Burel data using a module previously described from that same dataset? This seems circular.**

We agree with the reviewer that this does seem circular, however, we think this is not an issue because of the way that the signature and the clusters were constructed, as well as the context in which we used the TB signature. Our clustering was done using unbiased, co-expression based unsupervised learning. The TB signature was identified using a differential expression analysis. While the clusters and the TB gene signature were derived from the same dataset, they were both constructed from very different methods. Furthermore, our goal was not to create rigorous clusters from the dataset, but to demonstrate the usage of the metric on a disease dataset with a disease signature. We agree with the reviewer that the choice of this signature and dataset, while not inappropriate, could be seen as circular if the aim was to create publishable clusters from the Burel dataset.

**L340 “We selected the most representative of those iterations”
What does this mean? A better description of how the final gene sets were chosen should be included here or in the methods, this would be relevant to using the approach in other datasets.**

This refers to the pairwise comparison of gene-pairs between all 10 iterations of the k91 gene clusters. One of the 10 iterations had to be chosen to serve as our “optimal” set of clusters. The reliance on the pairwise comparison of gene-pairs, and why this was deemed appropriate, is now added in line 225-229.

**L281 More description should be included (in the methods) about how random gene sets were generated. Ie how many randomized gene sets, how large, are they non-overlapping? Etc.**

We thank the reviewer for pointing out this gap. We have added an additional comment detailing the random ground truth set at lines 153-155.

**L359-375 It is not clear (or stated) in which dataset this evaluation is being conducted.**

**L359-375 It is not clear in which RNASeq dataset these comparisons are being made. I would assume these comparisons are made using the Burel dataset (since a CD4 TB signature would not be expected in the healthy donor DICE data), but this is not stated.**

We apologize, the comparisons are being made between the k91 DICE clusters derived from the DICE dataset and the blood transcription modules (BTMs) derived from various disease and healthy datasets from the Chaussabel et. al. 2008 manuscript.

**Figures/Tables
Supplementary tables should more appropriately refer to biological “sex” as opposed to “gender”, consistent with terminology used throughout the manuscript.**

We thank the reviewer for catching this mistake, mentions of “gender” as a column heading in Supplementary Tables have been changed to “sex” to reflect the terminology from the rest of the manuscript.
